# Inhaled Corticosteroids Influence Pulmonary Microbiota in Severe Equine Asthma

**DOI:** 10.3390/ani16131994

**Published:** 2026-06-28

**Authors:** Estelle Manguin, Robert P. Dickson, Juliette Jamon, Valérie Dubuc, Mathilde Leclère

**Affiliations:** 1 Department of Clinical Sciences, Université de Montréal, St-Hyacinthe, QC J2S 2M2, Canada; estelle.manguin@vet-alfort.fr (E.M.); juliette.jamon@clinique-nac.com (J.J.); vdubuc@meuneriemondou.com (V.D.); 2 Division of Pulmonary and Critical Care Medicine, Department of Internal Medicine, University of Michigan, Ann Arbor, MI 48109, USA; rodickso@med.umich.edu

**Keywords:** asthma, corticosteroids, horses, microbiome, *16S rRNA* gene

## Abstract

The use of inhaled corticosteroids (ICs) could influence the respiratory microbiota. In animals with asthma it is, however, difficult to separate the immunomodulatory effects of ICs from their indirect effects via improvement of ventilation. Our objective was to determine if ICs alter the pulmonary microbiota independently from their effects on lung function, using a blinded, controlled trial in an experimental model of asthma exacerbation in horses. We treated horses with severe asthma with either bronchodilators alone, or in combination with ICs. Twelve horses in exacerbation received long-acting β2-agonist (LABA, salmeterol) or ICs/LABA (fluticasone/salmeterol) by inhalation, for 2 weeks. Lung function and bronchoalveolar lavages (BAL) were performed before and after treatment. *16S rRNA* gene quantification and sequencing were performed on BAL fluid, using digital droplet PCR and the Illumina MiSeq platform. Data were processed using the software package mothur v. 1.44.2. In the LABA group, pulmonary bacterial load and the relative abundance of Actinobacteria and Verrucomicrobia phyla decreased with treatment (*p* < 0.05 for both), and β-diversity differed from baseline (*p* = 0.007). The relative abundance of families and genera belonging to the Bacteroidetes phylum increased with ICs/LABA (*p* < 0.05). Lung function significantly improved with both treatments, suggesting that treatment-related differences in pulmonary microbiota could be attributed in part to medication, not solely to change in ventilation. However, it is not clear if these changes are positive or detrimental to the lung environment. Furthermore, lung function following treatment was not perfectly identical between groups.

## Simple Summary

In animals with asthma, it is difficult to separate the immunomodulatory effects of inhaled corticosteroids from their indirect effects via improvement of ventilation. This study investigated the pulmonary microbiota of horses with severe equine asthma treated with either bronchodilators alone or in combination with inhaled corticosteroids. Our objective was to determine if inhaled corticosteroids alter the pulmonary microbiota independently from their effects on lung function. Bacterial load and the relative abundance of some taxa did not change in the same way with both treatments. Since lung function significantly improved in both groups, this suggests that treatment-related differences in pulmonary microbiota could be attributed in part to medication, not solely to change in ventilation. However, it is not clear if these changes are positive or detrimental to the lung environment.

## 1. Introduction

The lungs contain a low but diverse microbial biomass composed of bacteria, fungi, and other micro-organisms. The role of bacterial infection or colonization in the development of asthma is uncertain, but it could be a contributing factor to clinical exacerbations [1]. The pulmonary inflammation induced by antigen inhalation and the associated bronchoconstriction could change the local environment of the lungs (decreased oxygen tension, lower pH, increased mucus), promoting bacterial growth of some taxa relative to others. This dysbiosis could in turn stimulate the local immune response and contribute to pulmonary inflammation. In people and in horses, the pulmonary microbiota is influenced by several factors, such as disease [2], the environment [3], and medication [4,5,6]. In people with asthma, changes in the composition of the pulmonary microbiota are associated with pulmonary function and lung inflammation [7], as well as with corticosteroids resistance [8].

Corticosteroids are widely used to treat asthma since they are effective at controlling airway inflammation and improving pulmonary function [9]. Corticosteroids can induce systemic as well as local immunosuppression within the respiratory tract [10,11]. Inhaled corticosteroids (ICs) could potentially influence pulmonary microbiota and promote the overgrowth of specific bacteria, which may in turn contribute to recrudescence of disease. Studies have also shown that corticosteroids can influence the pulmonary microbiota, by modulating relative abundances of different taxa, and influencing β-diversity or α-diversity [6,12,13,14]. Recently, such effects associated with ICs have been described in patients with chronic obstructive pulmonary disease (COPD) [15]. Alterations of microbiota have also been observed in the gastrointestinal tract of humans and animals receiving corticosteroids. These changes have been observed in subjects with and without inflammatory conditions; although, the effect seems more consistent across studies in which underlying inflammation is present. This suggests that corticosteroids affect the microbiota via decreasing local inflammation, but also that they can have other effects (by altering mucus production for example), or even some direct effects on steroid-metabolizing bacteria [16]. However, the mechanisms by which they act have not been clearly elucidated. They could have immunomodulatory-mediated effects; but they could also have ventilation-mediated effects, i.e., they could alter the microbiota mainly by restoring better ventilation, recreating a more normal local environment.

Severe equine asthma affects almost 15% of adult horses in northern regions [17], and is characterized by frequent episodes of reversible bronchoconstriction and airway inflammation. In genetically predisposed horses, exacerbations are induced by inhalation of allergens mostly present in hay (β-glucans, mites, endotoxins). Clinical exacerbations are characterized by wheezes; increased respiratory efforts, cough, and increased mucus. Exacerbations are also associated with an increase in pulmonary resistance and elastance, hypoxemia, and pulmonary inflammation (eosinophils, mast cells or neutrophils in the milder forms; predominantly neutrophils in the severe form). Clinical signs are controlled with corticosteroid administration, bronchodilators, and decreased exposure to antigens [18]. The impact of ICs on the pulmonary microbiota of horses with severe asthma is unknown.

The main objective of this controlled trial was to determine the relative contribution of ICs to changes in the pulmonary microbiota independently from their effects on lung function. To do so, we compared the pulmonary microbiota of horses treated with either bronchodilators (long-acting β2-agonist (LABA)) alone or in combination with ICs. We also compared the efficacy between treatments, with lung function data, blood gas analysis and BALF cytology. The hypothesis was that horses receiving ICs in addition to LABA would have greater modifications of their pulmonary microbiota than horses treated with LABA alone, despite similar improvement in lung function. The main results are part of the first author master’s dissertation [19] and some of the results of this study have been previously reported in the form of abstracts [20,21,22].

## 2. Materials and Methods

### 2.1. Horses

Twelve adult horses with severe asthma belonging to the Equine Asthma Research Laboratory were studied. Physiological data, history and housing are described in the Appendix A.1. The experimental protocol was performed in accordance with the Canadian Council on Animal Care guidelines and approved by the Animal Care Committee of the University of Montreal (protocol #18-Rech-1760, 29 November 2018).

### 2.2. Experimental Design

In this blinded, controlled trial, exacerbation was induced by exposure to dry hay when horses were housed together in the same barn. Once horses were in clinical exacerbation (labored breathing, coughing, lung resistance > 1 cmH_2_O/L/s), horses were ranked according to their lung function. The “odd” group consisted of horses 1 (highest resistance), 3 (3rd highest resistance), 5, 7, 9, 11, and the “even” group consisted of horses 2 (2nd highest resistance), 4, 6, 8, 10 and 12. Group allocation was then done by flipping a coin and the odd group received LABA. For 2 weeks, the ICs/LABA group received fluticasone and salmeterol (Advair^®^ 250 HFA MDI, GlaxoSmithKline, Brentford, UK, 2500/250 μg q12 h, *n* = 6), while the LABA group received salmeterol alone (Sigma-Aldrich Canada, Oakville, ON, Canada, 250 μg q8 h, *n* = 6). These dosages were expected to lead to similar improvement of ventilation based on previous studies [23,24,25]. Asthma clinical scores, tracheal mucus, lung function, BALF inflammation, and *16S rRNA* gene quantification and sequencing were assessed before and after treatment.

### 2.3. Clinical Scores, Tracheal Mucus Scores, and Lung Function

Asthma clinical scores were assessed by a person blinded to group allocation [26]. Tracheal mucus scores were assessed by a person blinded to time, horse identification and group allocation, based on anonymized endoscopic images [27]. Lung function was measured on unsedated horses, using an esophageal catheter and a pneumotachograph [28]. Gas exchange was assessed by arterial blood gas analysis sampled from the facial artery (arterial partial pressure of oxygen (PaO_2_) and carbon dioxide (PaCO_2_)). See also data Appendix A.

### 2.4. Sample Collection

Bronchoalveolar lavage fluid was collected on standing, sedated horses using a 2.5 m videoendoscope as previously described [29]. To avoid upper airway contamination, the endoscope was first advanced in a protective sheath until it reached the trachea [30]. It was then further advanced out of the sheath and into the right main bronchi until it lodged in a bronchus of 10 mm in diameter. Details about BALF collection and control specimens are provided in the Appendix A [31].

### 2.5. Sample Processing

Total deoxyribonucleic acid (DNA) was extracted from samples using the DNeasy Blood and Tissue Kit (Qiagen, Toronto, ON, Canada) following the manufacturer’s instructions, with minor modifications. The V4 region of the bacterial *16S rRNA* gene was amplified and sequenced using the Illumina MiSeq platform (Illumina Inc., San Diego, CA, USA) with a MiSeq Reagent Kit V2 (500 cycles) according to the manufacturer’s instructions with modifications for low-biomass samples. Bacterial *16S rRNA* gene was also quantified using digital droplet PCR (ddPCR). See also Appendix A [32,33].

### 2.6. Data Analysis

Sequence data were processed using the software mothur v.1.44.2, following the Standard Operating Procedure previously described [34,35]. Good quality reads were clustered in operational taxonomic units (OTUs) at the genus, family and phylum levels and classified according to the Ribosomal Database Project databank. Relative abundance, α and β diversity were analyzed, as well as linear discriminant analysis effective size (LEfSe, v. 1.0) [36] and correlations between sequencing data and function. Additional details are provided in Appendix A.

## 3. Results

### 3.1. Horses

**Figure 1:**
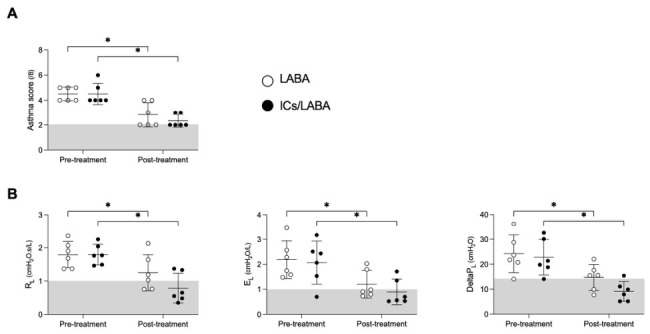
Asthma clinical scores and lung function. Asthma clinical scores (**A**) and lung function (**B**) in horses with severe asthma, before and after 2 weeks of treatment. Open circles: horses treated with LABA (*n* = 6). Solid circles: horses treated with ICs/LABA (*n* = 6). Black lines represent the mean and SD. Shaded area represents normal values in healthy horses. *: significant difference within the same group (*p* values for ICs/LABA and LABA: Scores: 0.024 and 0.039, RL: <0.0001 and 0.0057, EL: 0.0004 and 0.0013, deltaPL < 0.0001 and 0.0006). RL: lung resistance; EL: lung elastance; deltaPL: difference in pleural pressure; LABA: long-acting β2-agonist; ICs: inhaled corticosteroids.

There were no significant differences between groups for age, sex, and weight (Appendix A Table A1). As expected, horses were in clinical exacerbation with antigen exposure (increased respiratory efforts and cough). Treatments improved clinical scores in both groups (*p* = 0.024 and *p* = 0.039 in ICs/LABA and LABA group, respectively), without difference between groups before or after treatment (Figure 1).

### 3.2. Lung Function

**Figure 2:**
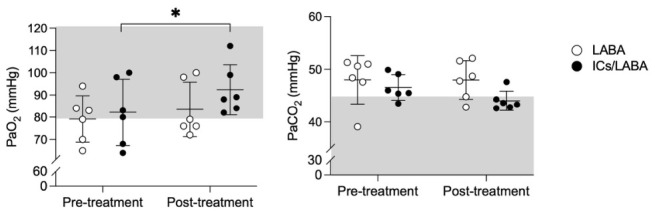
Arterial blood gas analyses. Arterial partial pressure of oxygen (PaO_2_) and arterial partial pressure of carbon dioxide (PaCO_2_) in horses with severe asthma before and after 2 weeks of treatment. Open circles: horses treated with LABA (*n* = 6). Solid circles: horses treated with ICs/LABA (*n* = 6). Black lines represent the mean and SD. Shaded area represents normal values in healthy horses. *: significant difference within the same group. LABA: long-acting β2-agonist. ICs: inhaled corticosteroids.

Horses developed airway obstruction with antigen exposure, characterized by lung resistance greater than 1 cmH_2_O/L/s. Treatments improved lung resistance, lung elastance, and difference in pleural pressure in both groups (*p* < 0.0001, *p* = 0.0004, and *p* < 0.0001 with ICs/LABA group; *p* = 0.0057, *p* = 0.0013, and *p* = 0.0006 in LABA group) (Figure 1). There were no significant differences between groups, but while 4 to 5 horses had normal resistance (4), elastance (4), and difference in pleural pressure (5) at the end of the ICs/LABA treatment, it was only the case for 2 horses for resistance, 4 for eleastance and 2 for difference in pleural pressure with LABA (see discussion). Five horses were hypoxemic and 10 were hypercapnic (PaO_2_ < 80 mmHg, PaCO_2_ > 45 mmHg) during exacerbation. PaO_2_ increased in 4 and 5 horses receiving LABA and ICs/LABA, respectively. This increase was significant in the ICs/LABA group only (*p* = 0.028), and while arterial blood gas analyzes did not differ between groups, all six horses had PaO_2_ above 80 mmHg at the end of the ICs/LABA treatment, it was only the case for two horses with LABA (Figure 2).

### 3.3. Lung Inflammation

**Figure A1:**
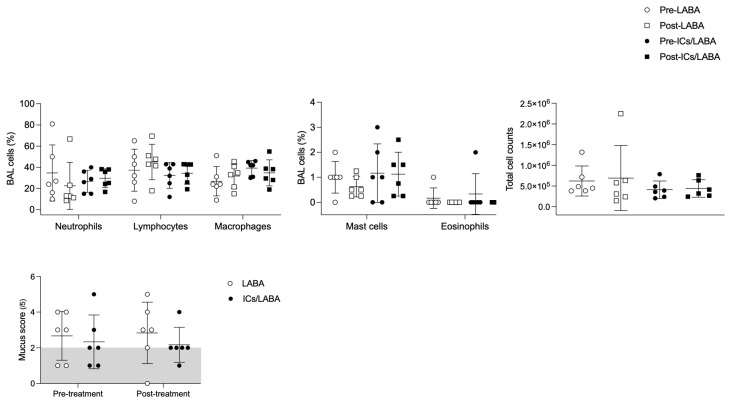
Bronchoalveolar lavage cytology and mucus scores. Bronchoalveolar lavage cell differential and total count and mucus scores in horses with severe asthma before and after 2 weeks of treatment. Open circles and squares: horses treated with LABA (*n* = 6). Solid circles and squares: horses treated with ICs/LABA (*n* = 6). Black lines represent the mean and SD. Gray area represents normal mucus scores in healthy horses. BAL: bronchoalveolar lavage. LABA: long-acting β2-agonist. ICs: inhaled corticosteroids.

As expected, horses developed lung inflammation with antigen exposure, characterized by bronchoalveolar lavage neutrophilia (neutrophils ≥ 10%). There was no significant effect of treatment in either group, which is not unexpected in the absence of environmental change (Appendix A Figure A1).

### 3.4. Bacterial DNA Load

**Figure A2:**
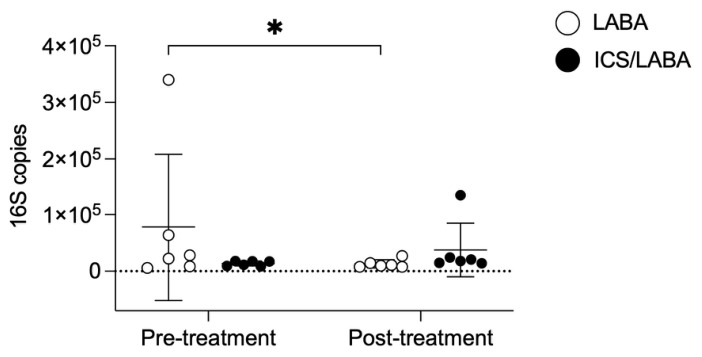
Bacterial *16S rRNA* gene quantification. *16S rRNA* gene quantification by digital droplet PCR in horses with severe asthma before and after 2 weeks of treatment. Open circles: horses treated with LABA (*n* = 6). Solid circles: horses treated with ICs/LABA (*n* = 6). Black lines represent the mean and SD. *: significant difference within group. LABA: long-acting β2-agonist. ICs: inhaled corticosteroids.

There was a small but significant reduction in *16S rRNA* gene burden after LABA treatment only (*p* = 0.0496; Appendix A Figure A2), with no significant difference between groups.

### 3.5. Sequencing Results

#### 3.5.1. Procedural Contaminants

Taxa detected in control specimens were significantly distinct from those detected in BALF specimens (*p* < 0.001, analysis of molecular variance (AMOVA)). The five most abundant OTUs detected in control specimens (47.8% of all sequences), were classified as *Pseudomonas* (OTU1), *Flavobacterium* (OTU2 and OTU3), *Bacillus* (OTU5), and an unclassified *Enterobacteriaceae* (OTU6). Collectively, these OTUs represented 23.4% of sequences detected in BALF specimens, with OTU2 and OTU3 representing 14.3% and 7.4% respectively. Since these OTUs, belonging mostly to the *Flavobacterium* and *Pseudomonas* genera, could represent environmental and procedural contaminants, we repeated α- and β-diversity analyzes by excluding these two genera. Since we found no major differences between analyses with or without these genera, we elected to keep all OTUs in the final analysis.

#### 3.5.2. Number of Reads

A total of 865,858 reads passed the quality filters and were retained for analysis from a total of 1,163,783 reads. A subsample of 4132 reads per sample was used for α-diversity analysis to decrease potential bias caused by nonuniform sample sizes. Coverage after subsampling and removing sequences found twice or less was on average 99.9%.

#### 3.5.3. β-Diversity: Membership and Structure at the Genus Level Between Samples

**Figure 3:**
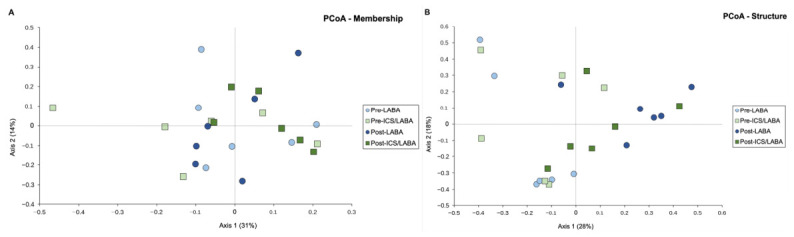
Principal coordinate analysis of bronchoalveolar lavage bacterial communities at the genus level. Bidimentional representation of the principal coordinate analysis (PCoA) of bacterial communities’ membership using the Classic Jaccard index (**A**) and structure using the Yue and Clayton index (**B**). Light blue and light green: before treatment. Dark blue and dark green: after 2 weeks of treatment. LABA: long-acting β2-agonist. ICs: inhaled corticosteroids.

β-diversity (membership and structure) was compared between groups with principal coordinate analysis (PCoA) plots and AMOVA test at the genus level. In terms of membership (that takes into account the different taxa), BALF samples did not cluster significantly by groups or time points (i.e., pre-post treatment) (Figure 3A). In contrast, the structure (that takes in account the different taxa and their relative abundance) of the BALF the samples differed significantly between pre- and post-treatment in the LABA group (light versus dark blue circles in Figure 3B, *p* = 0.007). This was not associated with a significant difference between groups before or after treatment (*p* = 0.678 and 0.176, respectively), or with a significant change between pre- and post- treatment in the ICs/LABA group (*p* = 0.054).

#### 3.5.4. Relative Abundance Analyzed Using the LEfSe Method at the Genus Level

**Figure 4:**
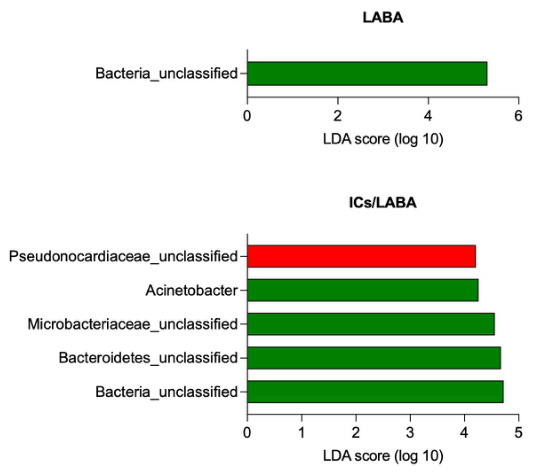
Linear discriminant analysis effect size (LEfSe). Genera in green were significantly overrepresented after treatment. Only one genus belonging to *Pseudonocardiaceae* was overrepresented before ICs/LABA treatment (in red). LABA: long-acting β2-agonist. ICs: inhaled corticosteroids. LDA: logarithmic discriminant analysis.

The difference in relative abundance at the genus level was analyzed using LEfSe (Figure 4). This method combines statistical significance with biological consistency and effect size estimation, while allowing for comparison of low-abundance genera. Bacteria unclassified at the genus level were overrepresented after both treatments. Three other genera were significantly overrepresented after ICs/LABA treatment: members of Bacteroidetes phylum, *Acinetobacter* (Proteobacteria phylum, *Moraxellaceae* family) and genera belonging to *Microbacteriaceae* family (Actinobacteria phylum).

#### 3.5.5. Analyses of Specific Taxa: Relative Abundance of the Most Common Phyla, Families, and Genera

**Figure 5:**
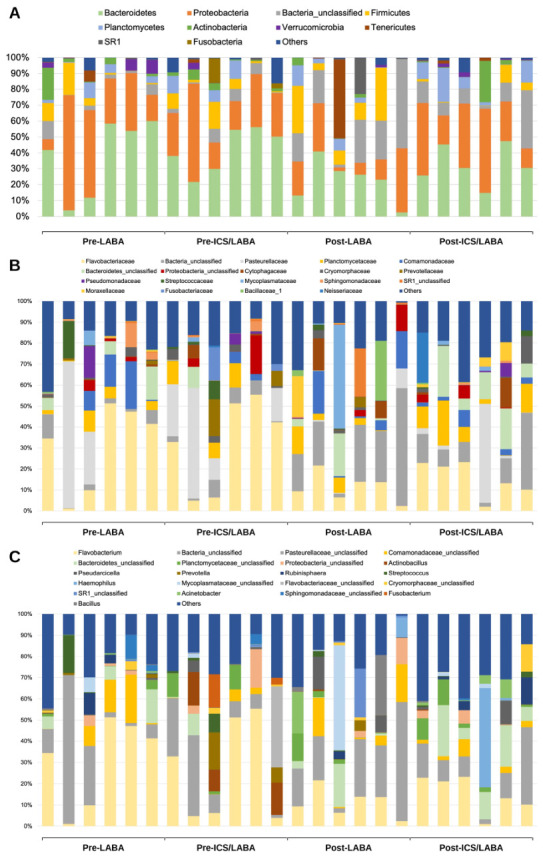
Relative abundance of predominant phyla, families, and genera. The 10 most common phyla (**A**), the 19 most common families (**B**), and the 21 most common genera (**C**) found in the bronchoalveolar lavage fluid of horses with asthma are represented. In panel (**A**), Actinobacteria (medium green) decreased after treatment with LABA. LABA: long-acting β2-agonist. ICs: inhaled corticosteroids.

The relative abundances of the most common phyla, families, and genera are presented in Figure 5. Sequences were classified into 23 different phyla, four of them accounting for 78.4% of the total number of sequences. The majority of OTUs were assigned to the Bacteroidetes (on average 35.8%) and the Proteobacteria phyla (29.9%), followed by the Firmicutes (6.7%) and Planctomycetes phyla (6.0%). Bacteria that were unclassified at the phylum level represented 11.6% of sequences.

**Figure 6:**
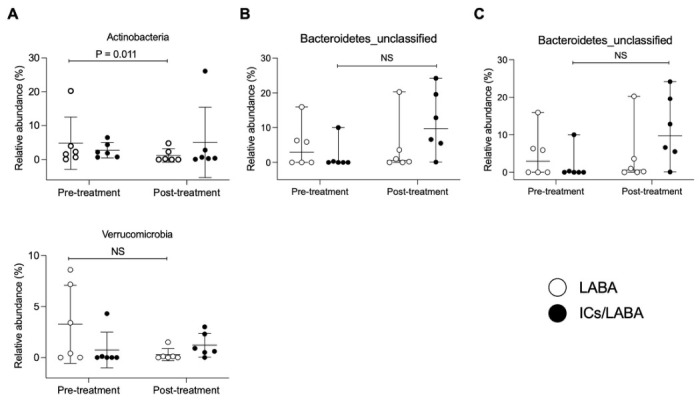
Relative abundance of predominant bacteria at the phylum (**A**), family (**B**), and genus (**C**) levels. Open circles: Bronchoalveolar lavage fluid from horses treated with LABA (*n* = 6). Solid circles: BALF from horses treated with ICs/LABA (*n* = 6). Black lines represent the mean and SD. NS: not significant after Benjamini–Hochberg correction. LABA: long-acting β2-agonist. ICs: inhaled corticosteroids.

Among the 10 most common phyla (relative abundance > 1%), no significant differences were found between groups, before or after treatments. However, treatment with LABA was associated with a decrease in Actinobacteria (4-fold decrease, *p* = 0.011) and Verrucomicrobia phyla (11.5-fold decrease, *p* = 0.025, not significant after correction), which was not observed with ICs/LABA (Figure 6A). The 19 most common families (>1% abundance) found in BALF samples together represented 82.3% of all sequences. Aside from the bacteria unclassified at the family level, the four most common families were *Flavobacteriaceae*, *Pasteurellaceae*, *Planctomycetaceae*, and *Comamonadaceae* (representing overall 24.2%, 10.2%, 6.0%, and 5.3% of all sequences, respectively). Among the 19 most common families, Bacteroidetes unclassified at the family level were relatively enriched following ICs/LABA treatment (6.7-fold increase, *p* = 0.034, not significant after correction). Except for these two families, no significant differences in the relative abundance of common families were found between groups and time-points (Figure 6B). The 21 most common genera (>1% abundance) found in BALF samples represented together 77.3% of all sequences. Aside from the bacteria unclassified at the genus level, the four most common genera were *Flavobacterium*, *Pasteurellaceae* unclassified at the genus level, *Comamonadaceae* unclassified at the genus level, and Bacteroidetes unclassified at the genus level (representing overall 22.8%, 6.6%, 5.2%, and 4.9% of all sequences, respectively). Among these 21 most common genera, Bacteroidetes unclassified at the genus level were relatively enriched after ICs/LABA treatment, as they were at the family level (same fold increase and *p* value, Figure 6C).

#### 3.5.6. α-Diversity: Richness and Diversity Within Samples

**Figure 7:**
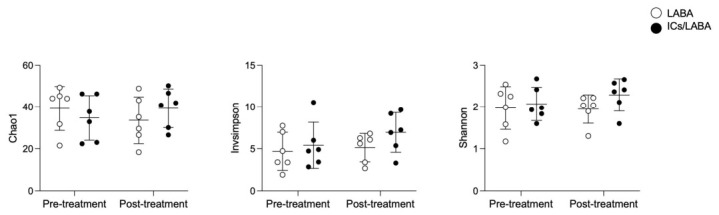
Indices of α diversity at the genus level of taxonomy. Open circles: BALF from horses treated with LABA (*n* = 6). Solid circles: Bronchoalveolar lavage fluid from horses treated with ICs/LABA (*n* = 6). Black lines represent the mean and SD. LABA: long-acting β2-agonist. ICs: inhaled corticosteroids.

The Chao richness estimator, the Inverse Simpson index, and the Shannon diversity index were used for characterization of α-diversity at the genus level of taxonomy. No significant changes were found between groups or time-points (Figure 7).

#### 3.5.7. Correlations Between Sequencing, Bacterial Quantification, Lung Function and Inflammation

During exacerbation (pre-treatment), some markers of severity were associated with a decrease in diversity or an increase in bacterial load (Inverse Simpson index/nasal score: r = −0.59, *p* = 0.045; ddPCR/PaCO_2_: r = 0.59, *p* = 0.049). After treatment, lower BALF neutrophilia was strongly associated with a decreased bacterial load (r = 0.85, *p* = 0.001, horses from both groups analyzed together).

## 4. Discussion

### 4.1. Inhaled Corticosteroids Influence the Pulmonary Microbiota Independently of Their Effects on Lung Function

This study investigated the pulmonary microbiota of horses with severe equine asthma housed together and treated with either LABA alone or in combination with ICs. The results support previous findings that the pulmonary microbiota is influenced by bronchodilators and ICs [6,12,13,14], and expand these findings by confirming that this occurs even in the absence of environmental or dietary confounding factors. Furthermore, the differences observed between the ICs/LABA and LABA groups suggest that ICs can alter the pulmonary microbiota not solely by improving lung function. With LABA alone, there was a *decrease* in bacterial load, a *decrease* in the relative abundance of Actinobacteria and Verrucomicrobia phyla, and a *modified* β-diversity. These changes could correspond to what can be expected from improving lung function and ventilation, without the anti-inflammatory effects of glucocorticoids. The improvement of lung function in the group treated with the combination ICs/LABA was associated with an *increase* in the relative abundance of some taxa (*Acinetobacter* genus, taxa belonging to Actinobacteria and Bacteroidetes phyla), with *no effect* on bacterial load and β-diversity. This could suggest that the effects observed with LABA are “blunted” or counteracted by the effects of ICs. Interestingly, an increase in members of Bacteroidetes phylum was also observed in the trachea of horses treated with dexamethasone administered intramuscularly [37], and people with asthma receiving ICs had greater abundance of *Acinetobacter* genus compared to those without ICs treatment [13].

In addition to *Acinetobacter* mentioned above, ICs are reported to modulate the relative abundances of different taxa in people with asthma [5,13,14]. A change in β-diversity with ICs treatment was also reported [14], but is not always observed [5], and neither of these studies by Huang and colleagues observed changes in α-diversity or bacterial load with inhalation of corticosteroids [5,14]. In horses with moderate asthma, nebulized dexamethasone was associated with decreased diversity of the nasal microbiota but not with changes in the α or β diversity of the tracheal microbiota [38]. Conversely, in another study by the same group, dexamethasone administered intramuscularly for 10 days affected the relative abundance of 11 OTUs from the tracheal microbiota of horses with and without asthma, but had no effect on their nasal microbiota [37]. Interestingly, the nebulization of low-dose dexamethasone does not improve lung function of horses with asthma, but dexamethasone administered systemically does, even at low doses [11,39,40], which suggests that nebulized dexamethasone mainly deposits in the upper airways of horses, and affect the microbiota locally. These studies in horses suggest that corticosteroids can alter the airway microbiota not only by improving ventilation but also more directly or locally, similarly to the findings of the current study.

### 4.2. Potential Mechanisms of Action of Corticosteroids on Pulmonary Microbiota

The mechanisms by which ICs influence the pulmonary microbiota have not been clearly elucidated. As mentioned above, they could act via their immuno-modulatory and anti-inflammatory effects [37,41], or they could mainly improve lung function, and consequently alter the local microbiota [42]. Alternatively, ICs treatment could exert a selective pressure favoring the growth of bacteria capable of metabolizing corticosteroids [5]. Steroid-metabolizing bacteria may also contribute to microbiota shifts. Members of Actinobacteria and Proteobacteria phyla can catabolize steroid ring structures such as the ones found in the membranes of eukaryotic cells, bile acid, steroid hormones, and their capacity to transform steroid compounds can vary with the aerobic/anaerobic conditions. Although mainly described in the gut, such mechanisms may exist in the airway [43,44]. In this study, the lack of decrease in bacterial load following ICs/LABA treatment, contrary to LABA treatment alone, favors the hypothesis that ICs could promote bacterial growth of some taxa to the detriment of others. This assumption is also supported by the results of the relative abundance and LEfSe analyzes, which highlighted taxa overrepresented after ICs/LABA treatment. However, this remains a hypothesis since microbial metabolomics and functional metagenomics were not evaluated. It is not possible based on these data to conclude whether these effects are positive or detrimental. One the one hand, corticosteroids have been implicated in the development of secondary pneumonia in human patients with asthma or COPD [41] and macrolides have been used in addition to bronchodilators and corticosteroids to improve lung function in asthmatic patients [45], but, on the other hand, there is still a debate on the role of causal relationship between corticosteroids and pneumonia [46,47]. Notably, in horses with severe asthma, who frequently receive oral or injectable corticosteroids for long periods of time (due to the high cost of inhaled medication), the development of secondary pneumonia remains anecdotal [11]. However, pneumonia and bacterial overgrowth are not systematically screened for and reported in equine medicine, and bacterial overgrowth should be considered in horses with poor response to corticosteroids therapy. ICs-associated shifts could predispose to secondary infections, with the enrichment of taxa such as *Acinetobacter*, a genus that has been associated with opportunistic infections and antimicrobial resistance [48], but it could also have beneficial effects (reduced inflammation, ecological stabilization) [49,50]. Finally, recent studies also reported effects of ICs on the pulmonary mycobiota and showed imbalances of trans-kingdom network between fungal and bacterial microbiota in human with asthma [13,14]. Our data and others could lead to recommendations for microbiota monitoring during long-term corticosteroids therapy, prior to the development of secondary infections.

### 4.3. Influences of Lung Function and Inflammation on Microbiota

In people, asthma exacerbations were associated with decreased pulmonary bacterial load and decreased α-diversity indices [2,6]. However, another study reported an increase in α-diversity, a negative correlation between bacterial load and respiratory hyperreactivity, and no change in global bacterial load [7]. Horses in exacerbation of asthma presented an increase in richness compared to the same horses in remission, but there was no change in diversity or bacterial load [30]. Here, a more severe exacerbation (based on PaCO_2_ and nasal score) was associated with an increased bacterial load and a decreased α-diversity, respectively. After treatment, a lower bacterial load was also associated with lower lung inflammation.

### 4.4. Strengths and Limitations of the Study

One limitation is the small sample size, which could have limited our ability to detect differences in the microbiota of the two groups. However, the fact that horses were housed in the same barn, received the same diet, and were turned out together limited the confounding variables brought by diet and environmental management [30]. In addition, the experimental design allows comparisons within the same animals, which improves statistical power. Another limit of this study included the fact that bronchodilators could have mild direct effects on the microbiota [51], and not only secondary to the improvement of lung function. In addition, the experimental model is not perfect, since horses receiving ICs/LABA had a slightly better ventilation than horses receiving only LABA. Strengths of this study include the numerous controls collected during sample collection, extraction, and throughout sequencing, as well as the technique used to collect BALF samples to limit upper respiratory tract contaminations. Contamination from equipment, environment or from commercial kits is an important issue in low-biomass microbiota analysis, such as the ones from bronchoalveolar lavage fluid. Here, *Flavobacterium* and *Pseudomonas* genera could represent true environmental or procedural contaminants, but they could also be part of pulmonary microbiota, and removing them could falsely increase relative abundances of other bacteria. Currently, there is no clear consensus on how to manage potential contaminations in microbiome analysis. We elected to re-run analysis with and without these OTUs and concluded that the results were not affected by their presence. Also, the relatively large number of unclassified bacteria could limit the detection of differences between communities. Potential solutions include shotgun metagenomics or long-read sequencing of the *16S rRNA* gene, with the latter being still limited by current incompleteness of reference databases for equine bacterial taxa [52]. Finally, *16S rRNA* gene sequencing does not provide direct information on metabolic functions, immunological pathways, microbial metabolomics, and functional metagenomics. The *16S rRNA* gene functional profiling using inference tools (PICRUSt2 and others) could have been used but can lack sensitivity especially with small numbers of subjects [53].

### 4.5. Future Directions

To investigate if the bacteriome recovers after ICs treatment, it would be interesting to compare the pulmonary microbiota of horses treated with either medication or allergen avoidance. In addition, it would be of interest to analyze the duration of microbiota alteration following treatments. In a larger study, it would also be relevant to include lung function (resistance or oxygenation) as a covariate in microbiota analyses to help account for disease severity. Finally, mycobiota, metagenomics and functional pathway analyses are still poorly described in horses and deserve further study.

## 5. Conclusions

The pulmonary microbiota is influenced by treatments and the differences observed between the ICs/LABA and LABA groups suggest that the changes in bacterial communities could be attributed in part to medication, not solely to change in ventilation. However, it is too early to determine if these changes associated with ICs are positive or detrimental to the lung environment.

## Data Availability

The data supporting the findings of this study is openly available in the UdeM Dataverse repository at URL https://doi.org/10.5683/SP4/CXPK4W, Borealis V1, accessed on 27 June 2026.

## Abbreviations

The following abbreviations are used in this manuscript:

AMOVA	analysis of molecular variance
BALF	bronchoalveolar lavage fluid
COPD	chronic obstructive pulmonary disease
ddPCR	digital droplet polymerase chain reaction
DNA	deoxyribonucleic acid
ICs	inhaled corticosteroids
LABA	long-acting β2-agonist
LEfSe	linear discriminant analysis effective size
OTU	operational taxonomic unit
PaCO_2_	arterial partial pressure of carbon dioxide
PaO_2_	arterial partial pressure of oxygen
PCoA	principal coordinate analysis
rRNA	ribosomal ribonucleic acid

## Appendix A

### Appendix A.1. Materials and Methods Supplement

#### Appendix A.1.1. Horses

Seven mares and five geldings, from 12 to 28 years of age (mean ± SD: 16.8 ± 5.5 years) and weighing 531 ± 56 kg were studied. There were four Quarter Horses, six Quarter Horses-associated breeds, one Thoroughbred and one Canadian Horse. They had a well-characterized history of reversible episodes of airway obstruction and inflammation with stabling and hay exposure. Physical examination and complete blood counts were performed before the study to exclude concurrent conditions such as respiratory infections. Throughout the study duration, all horses were housed in the same barn, on wood shavings, fed the same hay and concentrates, and turned out daily together in a paddock for 2 to 4 h.

#### Appendix A.1.2. Treatment Modalities

Fluticasone/salmeterol combination was administered by means of an equine aerosol chamber (AeroHippus, Trudell Medical International, London, ON, Canada), following the manufacturer instructions. Salmeterol powder was solubilized in sterile phosphate-buffered saline (PBS) at a concentration of 250 μg/mL, using Tween20 (Sigma-Aldrich Canada, Oakville, ON, Canada), as previously described [25], and administered using a face-tight mask equipped with a mobile ultrasonic nebulizer (SaHoMa, NEBU-TEC International, Elsenfeld, Germany).

#### Appendix A.1.3. Asthma Clinical Scores

Nasal and abdominal components were added to obtain a combined clinical score ranging from 2 to 8, as previously described [26]. Nasal flaring was scored from 1 to 4: 1 = no flaring, 2 = slight, occasional flaring of nostrils, 3 = moderate flaring of nostrils, and 4 = severe continuous flaring of nostrils. The abdominal component of breathing was scored from 1 to 4: 1 = no abdominal component, 2 = slight abdominal movement, 3 = moderate abdominal movement, and 4 = severe, marked abdominal movement.

#### Appendix A.1.4. Lung Function

Standard lung mechanics were recorded on standing, unsedated horses, as previously described [28]. Briefly, transpulmonary pressure was estimated by means of an esophageal catheter, and airflow was measured using a heated pneumotachograph connected to a face-tight mask placed on the horses’ nose. Pulmonary resistance and elastance were then computed using dedicated software (Labdat/Anadat program on MS-DOS, and FlexiWare software 7.6, SCIREQ, Montreal, QC, Canada). A single-compartment linear model of the lung was employed, expressed as: PL = (EL × VT) + (RL × $\dot{V}$F) + k, where PL: transpulmonary pressure, EL: pulmonary elastance, VT: tidal volume, RL: pulmonary resistance, $\dot{V}$F: respiratory flow, and k: transpulmonary end-expiratory pressure. Arterial blood was sampled prior to the administration of sedative, using minimal restraint while the horses were in their stalls and breathing ambient air. Samples were collected anaerobically into pre-heparinized syringes (Arterial Blood Gas Sampler 9025TRU, Vyaire Medical Inc., Mettawa, IL, USA) by direct needle puncture of the transverse facial artery. Between 0.5 and 1 mL of blood was collected and analyzed immediately using a portable automated blood gas analyzer (i-STAT, Abbott Point of Care, Abbott Laboratories, Chicago, IL, USA). Partial pressure of oxygen (PaO_2_) and partial pressure of carbon dioxide (PaCO_2_) corrected for the patient body temperature were recorded.

#### Appendix A.1.5. Bronchoscopy and Tracheal Mucus Score

Bronchoscopy was performed on standing, sedated horses (detomidine (Dormosedan, Zoetis, Parsippany, NJ, USA) 0.008–0.01 mg/kg IV, and butorphanol (Torbugesic, Zoetis, Florham Park, NJ, USA) 0.01–0.02 mg/kg IV) using a 2.5 m fiber-optic videoendoscope (Olympus, GIF-H180, Olympus Canada Inc., Richmond Hill, ON, Canada). To avoid upper airway contamination, the endoscope was first advanced in a protective sheath through the nasal passages, the glottis and into the proximal trachea. Then the endoscope was further advanced out of the sheath as previously described [30]. The pictures taken were anonymized and scored by a blinded evaluator. Tracheal mucus accumulation was scored from 0 to 5 [27], with 0 corresponding to no mucus; 1 to little mucus accumulation in small blobs; 2 to moderate mucus accumulation; 3 to marked, stream forming mucus blobs; 4 to large, pool-forming accumulation; and 5 to extreme accumulation of mucus. A score greater than 2 was considered abnormal [18].

#### Appendix A.1.6. Bronchoalveolar Lavage

BALF was collected as previously described [29]. Briefly, the videoendoscope was lodged in a 10 mm bronchi after infusing 60 mL of sterile lidocaine 0.5%. Two boluses of 250 mL of sterile prewarmed isotonic saline (0.9% NaCl) were infused and quickly reaspirated. Samples were put immediately on ice and processed within 2 h. Exactly 15 mL of BALF were frozen at −80 °C for *16S rRNA* gene quantification and sequencing. Remaining BALF cells were centrifuged at 1600 rpm at 4 °C, washed twice in PBS 1X and total cell count was determined (ADAM automatic Cell Counter, Montreal-Biotech Inc., Montreal, QC, Canada). Amounts of 100 to 200 μL were used for cytopreparations (Rotofix32A, Hettich, Berlin, Germany) and stained with a modified Wright-Giemsa (DiffQuick, Fisher Scientific, Waltham, MA, USA). Differential cell count was performed on 400 nucleated cells, excluding epithelial cells. Between each horse, the endoscope and the protective sheath were cleaned with isopropyl alcohol (70%) and multi-enzymatic solution (Endozyme, Ecolab, Mississauga, ON, Canada), and then immersed for 10 min in a 1% potassium monopersulfate solution (Virkon^®^, 02125021, Vetoquinol Inc., Lavaltrie, QC, Canada) before being thoroughly rinsed with sterile water.

#### Appendix A.1.7. Control Specimens

Prior to each BALF procedure, 50 mL of sterile saline was instilled through the main channel of the endoscope, collected in sterile tubes, and processed as BALF samples (“scope rinse specimens”). Twenty reagent control specimens were also collected since reagents used in DNA isolation and library preparation can contain bacterial DNA [31]. Samples from the air from the barn (*n* = 3), the air from the biological safety cabinet (*n* = 2), lidocaine (*n* = 4) and sterile water (*n* = 4) were also collected, quantified, and sequenced.

#### Appendix A.1.8. DNA Extraction

Five ml of each sample was centrifuged at 14,000 rpm for 30 min at 4 °C and 360 μL of lysis buffer was added to cell pellets and transferred to 0.7 mm garnet bead tubes (VWR, Radnor, PA, USA). Samples were bead beaten for 1 min at the homogenize setting of a Mini-BeadBeater-8 (Biospec, Bartlesville, OK, USA) and centrifuged for 1 min at 14,000 rpm at room temperature. Samples were incubated for 1 h at 55 °C with proteinase K and 30 min at 70 °C with a guanidine hydrochloride buffer provided with the kit. We then continued with the DNeasy^®^ Blood and Tissue Kit protocol (Qiagen) starting with step 3 (DNA precipitation with ethanol). After DNA extraction step, 20 μL of each sample was sent to the University of Michigan for ddPCR and high throughput sequencing.

#### Appendix A.1.9. High Throughput Sequencing

Sequencing was performed using the MiSeq IEMFileVersion 4 platform (Illumina) with a MiSeq Reagent Kit V2 (500 cycles) according to the manufacturer’s instructions with modifications for low-biomass samples. The V4 region of the bacterial *16S rRNA* gene was amplified by PCR using published primers, as previously recommended [32]. Briefly, Accuprime High Fidelity Taq was used in place of Accuprime Pfx SuperMix (Thermo Fisher Scientific, Waltham, MA, USA). Primary PCR cycling conditions were 95 °C for two minutes, followed by 20 cycles of touchdown PCR (95 °C 20 s, 60 °C 20 s and decreasing 0.3 degrees each cycle, 72 °C 5 min), then 20 cycles of standard PCR (95 °C for 20 s, 55 °C for 15 s, and 72 °C for 5 min), and finished with 72 °C for 10 min.

#### Appendix A.1.10. Digital Droplet PCR

Bacterial DNA was quantified using a QX200 Droplet Digital PCR System (BioRad, Hercules, CA, USA). Primers and cycling conditions were performed according to a previously published protocol [33]. Specifically, primers were 5′-GCAGGCCTAACACATGCAAGTC-3′ (63F) and 5′-CTGCTGCCTCCCGTAGGAGT-3′ (355R). The cycling protocol was 1 cycle at 95 °C for 5 min, 40 cycles at 95 °C for 15 s and 60 °C for 1 min, 1 cycle at 4 °C for 5 min, and 1 cycle at 90 °C for 5 min all at a ramp rate of 2 °C/second. The BioRad C1000 Touch Thermal Cycler (BioRad, Hercules, CA, USA) was used for PCR cycling. Droplets were quantified using the Bio-Rad Quantisoft software, v.1.7 (BioRad, Hercules, CA, USA). Two replicates were used per sample.

#### Appendix A.1.11. Data Analysis

Sequence data were processed using mothur v.1.44.2, following the Standard Operating Procedure using a minimum sequence length of 250 base pairs, as previously described [34,35]. Good quality reads were clustered in operation taxonomic units (OTU; 97% identity) at the genus, family and phylum levels and classified according to the Ribosomal Database Project (RDP) databank. Sequences that were present ≤ 2 times were removed from analysis. For relative abundance and ordination analysis, samples were reported to the percent of total reads, and analysis was limited to taxa that represented more than 1% of the sample population; all taxa were included in the diversity analysis. Alpha-diversity was assessed by the Chao richness estimator, Inverse Simpson index, and Shannon index. The Jaccard index and the Yue and Clayton index were used to analyze beta-diversity by comparing community membership (that considers the different taxa) and structure (that considers the different taxa and their distribution within the community), respectively. Beta-diversity was compared using the analysis of molecular variance (AMOVA) tests and was also explored visually using principal coordinate analysis (PCoA). Differences between groups were further explored using linear discriminant analysis effective size (LEfSe, version 1.0) [36], which uses factorial Kruskal–Wallis sum-rank and a Wilcoxon rank-sum to detect features with biological significance by comparing the abundance in all populations, including those with low abundance. LEfSe uses linear discriminant analysis (LDA) to estimate the effect size of each differentially abundant feature. Alpha values for the factorial Kruskal–Wallis test among classes and for the pairwise Wilcoxon test between subclasses were set to 0.05. Threshold on the logarithmic LDA score for discriminative features was set to 2.0 and the strategy for multiclass analysis was set to all-against-all.

Statistical analyses for clinical score, lung function (standard lung mechanic, arterial blood gas), lung inflammation (tracheal mucus score, neutrophilia on BALF), bacterial load (*16S rRNA* ddPCR), relative abundances of major taxa at the phylum, family, and genus levels, alpha-diversity indices (Chao, Shannon, Inverse Simpson), and beta-diversity indices (membership, structure) were performed using GraphPad Prism 8.0.2, R version 4.0.3 and mothur v.1.44.2. Normality was assessed by visual inspection of the data and with the Agostino–Pearson and Kolmogorov–Smirnov tests. Data not normally distributed were transformed using arcsine square root transformation. Parametric (analysis of variance (ANOVA) with Benjamini–Hochberg corrections for taxa-level analysis) or non-parametric (Wilcoxon matched-pairs signed rank test, Mann–Whitney test, Mantel–Haenszel test, Cochran–Mantel–Haenszel test) tests were used when appropriate. Age, sex, and weight were compared between groups with Student’s *t* tests and Fisher’s exact tests. *p* values < 0.05 were considered significant. Spearman correlations were also done between sequencing data, bacterial quantification (ddPCR), lung function data, lung inflammation (percentage of neutrophils), and clinical scores before and after treatment.

#### Appendix A.1.12. Identification of Procedural Contaminants

As detailed above, control specimens were collected to identify the influence of procedural contamination. Specimens were processed in a randomized order to minimize the risk of false pattern formation due to reagent contamination. We compared the bacterial taxa detected in control specimens and in BALF using AMOVA and by comparing relative abundances between samples. No taxa were excluded from final analysis.

### Appendix A.2. Appendix Figures and Table

**Table A1:** Age, sex, breed, and weight of the 12 horses included in the study.

Horse	Treatment Group	Age (Years)	Sex	Breed	Weight (kg)
H1	LABA	28	Mare	QH-X	445
H2	LABA	15	Mare	QH-X	514
H3	LABA	12	Gelding	Canadian	646
H4	LABA	10	Gelding	QH	577
H5	LABA	19	Mare	QH-X	593
H6	LABA	14	Gelding	QH-X	499
Mean (SD)		16.3 (6.5)			545.7 (72.9)
H7	ICs/LABA	14	Gelding	QH	480
H8	ICs/LABA	15	Mare	QH-X	555
H9	ICs/LABA	18	Mare	QH	508
H10	ICs/LABA	27	Gelding	QH	520
H11	ICs/LABA	16	Mare	QH-X	559
H12	ICs/LABA	14	Mare	Tb	479
Mean (SD)		17.3 (5.0)			516.8 (35.0)
*p*-value		0.770	>0.999		0.403
Test between groups		Student’s *t* test	Fisher’s exact test		Student’s *t* test

LABA: long-acting β2-agonist. ICs: inhaled corticosteroids. QH: Quarter Horse. QH-X: Quarter Horses-associated breeds (Quarter Horse crossbreed, Paint Horse, Paint crossbreed). Tb: Thoroughbred.
